# Genomic Characterization of *Cryptococcus neoformans* Isolated from Zebra Dove Excreta in Songkhla, Thailand

**DOI:** 10.3390/vetsci12090827

**Published:** 2025-08-28

**Authors:** Saowakon Indoung, Sanicha Chumtong, Sakaoporn Prachantasena, Ratchakul Wiriyaprom, Komwit Surachat, Sarunyou Chusri, Rattanaruji Pomwised, Ruttayaporn Ngasaman

**Affiliations:** 1 Faculty of Veterinary Science, Prince of Songkla University, Songkhla 90110, Thailand; saowakon.i@psu.ac.th (S.I.);; 2 Department of Biomedical Sciences and Biomedical Engineering, Faculty of Medicine, Prince of Songkla University, Songkhla 90110, Thailand; 3 Translational Medicine Research Center, Faculty of Medicine, Prince of Songkla University, Songkhla 90110, Thailand; 4 Division of Biological Science, Faculty of Science, Prince of Songkla University, Songkhla 90110, Thailand

**Keywords:** genomic, *Cryptococcus neoformans*, whole-genome sequencing, zebra dove, Thailand

## Abstract

The research aimed to study the genome of *Cryptococcus neoformans* isolated from bird excreta. Thirteen isolates were cultured, colony stained, and underwent biochemical testing confirmation by nested polymerase chain reaction using ITS1-ITS4 and CN4-CN5 primers, respectively. Antifungal susceptibility testing and whole-genomic sequencing were analyzed. The results determined that all isolates were susceptible to amphotericin B (100%), fluconazole, and itraconazole (92.3%). One isolate (DOP3) showed resistance to fluconazole and itraconazole (MIC >64 and >8 µg/mL, respectively). A phylogenetic tree showed the identity of *C. neoformans* (serotype A). The genome of resistant (DOP3) and non-resistant isolates (DOP3.1) had 14 chromosomes. DOP3 consisted of 38 candidate antifungal resistance genes, which were the most active against azoles (14). The annotated genes in the azole group mostly were in the ATP-binding cassette transporter transmembrane superfamily. Resistance genes against FCZ were in the transcription factors (*HAP2, HAP5*), zinc finger (*NRG1*), cytochrome P450 (*ERG11*), and Myb-like DNA-binding domain (*REB1*). The most frequent resistance genes against ITZ were cytochrome P450 (*ERG5* and *ERG11*) and a transcription factor (*HAP5).* DOP3.1 also consisted of 26 candidate resistance genes against azoles. Resistance genes against the azole group belong to the *ABC* transporter transmembrane superfamily. Resistance genes against FCZ belong to cytochrome P450 (*ERG11*), the zinc finger (*NRG1*), and the CCAAT binding transcription factor (*HAP2*). Resistance genes belonging to cytochrome P450 (*ERG5*) were found against ITZ. This research provides the first report of *C. neoformans* (serotype A) in zebra dove excreta, drug susceptibility to a resistant strain, and identification of resistance genes. Farm sanitation should be strictly applied, and immunocompetent people should avoid contact with zebra dove excreta.

## Simple Summary

This research provides the first report of *Cryptococcus neoformans* (serotype A) in zebra dove excreta, drug susceptibility to a resistant strain, and identification of resistance genes. This research aimed to characterize the whole genome of *C. neofomans* recovered from zebra dove excreta in southern of Thailand. The antifungal susceptibility test demonstrated only one isolated resistance to fluconazole and itraconazole. That resistant isolate and a representative non-resistant isolate were chosen for whole-genome analysis to gain insight into their genomes. The results determined antifungal resistance phenotypes correlated with genotypes of both isolates. This research suggests that infection prevention should be prioritized as drug resistance is emerging in *C. neofomans*.

## 1. Introduction

*Cryptococcus neoformans* is an encapsulated basidiomycetous yeast classified into three variants with five serotypes: var. *grubii* (serotype A); var. *neoformans* (serotype D); var. *gattii* (serotypes B and C); and serotype AD (hybrid of serotypes A and D) [1]. The genus *Cryptococcus* consists of the *C. neoformans* species complex (CNSC) and the *C. gattii* species complex (CGSC). The CNSC has two species with five major molecular types: *C. neoformans* (VNI, VNII, and VNB, collectively called serotype A), *C. deneoformans* (VNIV, commonly called serotype D), and their hybrids (VNIII, serotype AD) [2]. Recently, five species were recognized in the CGSC according to genotype: *C*. *gattii* (VGI), *C*. *deuterogattii* (genotype VGII), *C*. *bacillisporus* (VGIII), *C*. *tetragattii* (VGIV), and *C*. *decagattii* (VGVI) [3]. The CNSC is distributed worldwide and causes systemic cryptococcosis in mostly immunocompromised patients, whereas the CGSC is often found in tropical and subtropical regions and affects immunocompetent individuals [4].

*C. neoformans* are mostly found in birds but are also seen in cats, dogs, cattle, horses, sheep, goats, and wild animals [5]. In birds, there have been reports of finding *C. neoformans* in pigeon excreta [6]. The prevalence of the CNSC and CGSC in pigeon excreta samples from Chiang Mai, Thailand, was 6.71% [7]. *Cryptococcus* spp. have also been detected by nested polymerase chain reaction (PCR) in 15% of zebra doves in southern Thailand [8]. *C. neoformans* in pet birds appears to behave as a primary pathogen in immunocompetent hosts, and it is normally found in soil contaminated with avian excreta and decaying vegetation [9,10,11]. The infection can be easily transferred when organisms inhale *C. neoformans* cells or basidiospores in the environment, especially as part of dust containing avian excreta [12,13]. Inhaled *C. neoformans* cells that cause human cryptococcosis, which affects the respiratory tract, central nervous system, eyes, and skin, can be deposited in the alveolae [14]. The disease causes meningitis and is usually fatal if untreated. Over 30 years ago, cryptococcosis was present in 5–10% of HIV-positive patients [15].

The global incidence and mortality due to cryptococcosis has been estimated as 957,000 cases per year (range 371,700–1,544,000 cases) and 181,000 deaths [16,17]. In Africa, the mortality rates due to *C. neoformans* in HIV patients were as high as 41–61% [18,19]. In Thailand, human cryptococcosis cases have been found to be the second leading infection after tuberculosis in HIV-infected, hospitalized patients with acute respiratory infections [20]. Interestingly, the trend of infection in the United States has changed to people who are not HIV-positive [21].

Successful treatment of *C. neoformans* has used amphotericin B (AMB), 5-fluorocytosine (5-FC), and various azoles such as fluconazole (FCZ) and itraconazole (ITZ) [22]. There are emerging strains exhibiting resistance to FCZ [23], AMB [24], 5-FC, and ITZ [25]. Therefore, our study aimed to analyze the antifungal susceptibility of *C. neoformans* recovered from zebra dove excreta collected in Songkhla province, Thailand. Moreover, our goal was to characterize the antifungal-resistant strains and elucidate the molecular characterization of the antifungal resistance gene by whole-genome sequencing. Our results will provide information on public health concerns to farmers and people who are in close contact with zebra doves.

## 2. Materials and Methods

### 2.1. Cryptococcus Neoformans ISOLATES

From a previous study [8], from June 2019 to April 2020, a total of 148 zebra dove dropping samples were collected from 14 farms in Chana district, Songkhla, Thailand. A total of 44 isolates were examined for characteristics and biochemical test results as *Cryptococcus* spp. and kept at −80 °C in glycerol stock tubes and subcultured on Sabouraud dextrose agar (SDA) (Oxoid, UK). However, only thirteen isolates were recovered from the stock and were included in this study. *Candida albicans* ATCC 90028 was used as quality control. The inoculum was prepared from 48-hour-old cultures suspended in 0.85% normal saline to 0.5 McFarland (1–5 × 10^6^ CFU/mL) measured by a spectrophotometer (absorbance = 530 nm) and diluted to 0.5–2.5 × 10^3^ CFU/mL in RPMI 1640 broth medium [26].

### 2.2. Antifungal Susceptibility Testing

Testing was performed by the broth microdilution method following Clinical and Laboratory Standards Institute guidelines [26] with some modification. The RPMI 1640 broth medium containing glutamine without bicarbonate (Gibco, NY, USA) was buffered to pH 7.0 by adding 0.165 mol/L 3-(N-morpholino) propanesulfonic acid (Sigma, MA, USA) and supplemented with 2% glucose. The RPMI 1640 broth medium was sterilized by passing it through a filter with a pore size of 0.22 mm before being used in the susceptibility tests. Antifungal powders of AMB (PanReac, Barcelona Spain), FCZ (Sigma, MA, USA), and ITZ (Sigma, MA, USA) were prepared at 100-fold concentration as stock solutions. The three antifungal products were tested in the following ranges: AMB at 0.02–16 μg/mL; FCZ at 0.06–64 μg/mL; and ITZ at 0.01–8 μg/mL.

**Figure 1:**
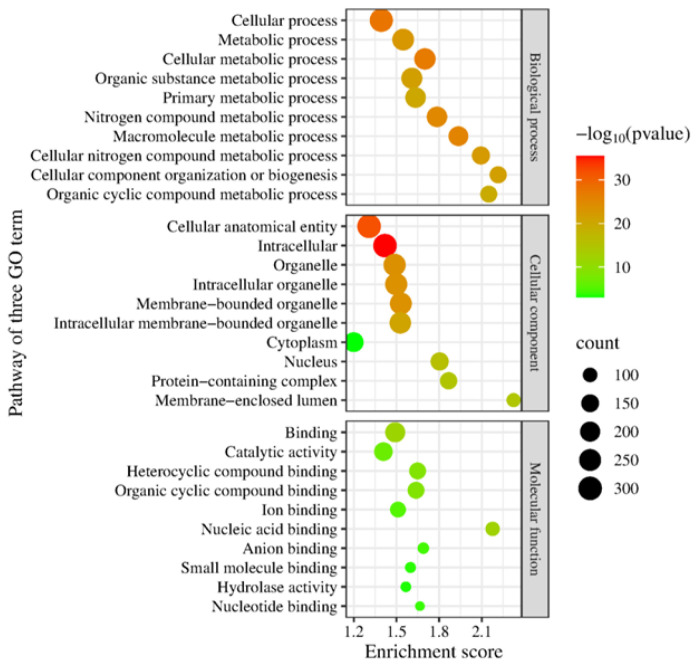
The top ten pathways of protein function annotation for three Gene Ontology (GO term) aspects, including biological processes, cellular components, and molecular function of *C. neoformans* DOP3 isolates.

Each plate well was filled with 100 μL of an antifungal dose followed by 100 μL of the *C. neoformans* inoculum. RPMI 1640 broth medium (100 μL) without any antifungal product served as the medium control, and RPMI 1640 broth medium (100 μL) with only *C. neoformans* inoculum was the growth control. The test was performed twice. The plates were incubated at 35 ± 2 °C for 48 h, after which 0.01% resazurin was added to the wells. The plates were incubated for another 3 h to measure the number of viable cells [27]. The minimal inhibitory concentration (MIC) was visually determined by pink/red to blue as the endpoint (Figure 1) after 72 h of incubation. The MIC endpoints for the antifungal products were AMB: susceptible (S) < 2 μg/mL, resistant (R) ≥ 2 μg/mL; FCZ: S ≤ 8 μg/mL, 8 μg/mL > susceptible-dose-dependent (SDD) < 16 μg/mL, R ≥ 16 μg/mL and ITZ: S ≤ 0.125 μg/mL, 0.25 μg/mL ≥ SDD ≤ 0.5 μg/mL, R ≥ 1 μg/mL [28]. After the MIC was determined, the wells that showed no visible *C. neoformans* growth was subsequently cultivated on SDA and incubated at 35 ± 2 °C for 24 h to determine the minimum fungicidal concentration (MFC).

### 2.3. DNA Extraction, PCR Confirmation, and Whole-Genome Sequencing

DNA from the *C. neoformans* isolates was extracted by using the Geneaid™ DNA Isolation Kit (Yeast) according to the manufacturer’s instructions. Pure DNA was used for nested PCR [29]. Briefly, the first step of amplification targeted the internal transcribed spacer (ITS) region by using two primers, ITS-1 (5′-TCCGTAGGTGAACCTGCGG-3′) and ITS-4 (5′-TCCTCCGCTTATTGATATGC-3′), to obtain an amplicon of approximately 500–600 bp. The second reaction used the amplicon with the targeting primers CN-4 (5′-ATCACCTTCCCACTATT CACACATT-3′) and CN-5 (5′-GAAGGGCATGCCTGTTT GAGAG-3′) to produce an amplicon of 136 bp. The PCR mixtures of both steps were run in a Bio-Rad Thermal Cycler (Bio-Rad Laboratories Inc., Hercules, USA) with the following protocols: heating at 94 °C for 5 min, followed by 35 cycles of denaturation at 94 °C for 30 s. each cycle, annealing at 55 °C for 45 s, and DNA extension at 72 °C for 1 min, with a final heating at 72 °C for 7 min. The genomic DNA was performed as a short-read whole-genome sequencing with the MGISEQ-2000 platform (MGI, Shenzhen, China) with 150 bp paired-end reads.

### 2.4. Genome Assembly and Genome Annotation

The raw reads (FASTQ format) were de novo assembled using SPAdes version 3.12 to generate genome contigs and scaffolds [30]. Geneious Prime version 2024.0.5 removed all contig-assembled sequences that were less than 200 bp. Two assembled sequences (FASTA format) were confirmed with the BUSCO version of Galaxy 5.5.0 plus galaxy0. The quality assessment results of the assembled genomes of the *C. neoformans* isolates used QUAST version 5.0.2 [31]. The assembled sequences are available at the National Center for Biotechnology Information (NCBI) BioProject ID PRJNA1211297. The BioSample ID is SAMN46272484 for *C. neoformans* DOP3 and SAMN46272485 for *C. neoformans* DOP3.1. The assembled sequences were annotated using AUGUSTUS (Galaxy version 3.4.0) (https://usegalaxy.org/, accessed on 1 May 2025). The *C. neoformans* (*cryptococcus*) model organism was used to predict gene locations and protein-coding genes for this study. The protein-coding sequence from AUGUSTUS was conducted with functional annotation. The EggNOG-mapper version 2.1.12 (http://eggnog-mapper.embl.de/, accessed on 1 May 2025) with default parameters was used to predict functional annotation against the database of EggNOG 5. The database has functional information from the Gene Ontology annotation source (GO annotation) [32]. To determine the functional roles of genes and their associated biological pathways, i.e., proteins in biological processes, molecular functions, and cellular components, we used ShinyGO version 0.81 [33] and selected the species *C. neoformans* with taxonomy ID 5207. From the ShinyGO results, we used the SRplot web server (https://www.bioinformatics.com.cn/en, accessed on 1 May 2025) to visualize the biological pathways of functional proteins [34].

### 2.5. Searching Candidate Antifungal Resistance Genes

The protein-coding sequences of *C. neoformans* DOP3 and DOP3.1 isolates from AUGUSTUS were conducted for prediction of antifungal resistance genes. The search for candidate antifungal resistance genes in *C. neoformans* used the ResFungi database and Hidden Markov Models (HMMs) specific for antifungal resistance genes with the percentage of identity and coverage ranged between 85.30 to 99.99. (ResFungi.hmm). The HMM repository contains profiles for fungal genes that have known mutations that confer resistance to antifungal drugs. The profiles contain information about genes with mutations that confer resistance to caspofungin (CAS), micafungin, FCZ, ITZ, and voriconazole. The program hmmsearch version 3.3.2 (http://hmmer.org/, accessed on 1 May 2025) was used to search for profiles against a sequence database.

### 2.6. Comparative and Phylogenetic Tree Analysis

The assembled sequences (FASTA format) of 230 previously published *Cryptococcus* genomes were taken from the NCBI database. Data displays the BioProject ID, BioSample, location, and isolate source information for all isolates. All assembled sequences from NCBI were annotated using AUGUSTUS (Galaxy version 3.4.0). A total of 232 protein-coding sequences from AUGUSTUS were analyzed for single-copy orthologous genes and aligned using OrthoFinder version 2.5.5 with default parameters [35]. The protein-coding sequences of isolates *C. neoformans* DOP3 and DOP3.1 were compared with 230 other isolates of *Cryptococcus* from the NCBI database, including *C. neoformans* (n = 184), *C. amylolentus* (n = 2), *C. wingfieldii* (n = 2), *C. floricola* (n = 2), *C. bacillisporus* (n = 2), *C. decagattii* (n = 1), *C. depauperatus* (n = 2), *C. deuterogattii* (n = 12), *C. gattii* (n = 9), *C. tetragattii* (n = 1), and *Cryptococcus* sp. (n = 13). The phylogenetic tree from OrthoFinder was visualized using Interactive Tree of Life version 7 (https://itol.embl.de/ accessed on 1 May 2025) [36]. We included 55 assembled sequences (FASTA format) from the NCBI database, as well as two additional assembled sequences. FASTA files (n = 57) were compared pairwise for average nucleotide identity (ANI). The ANI was evaluated using FastANI v1.32 [37].

## 3. Results

The 13 isolates on SDA for 3 d at 35 °C showed colonies that appeared as round cells with elevated and raised surfaces with smooth, mucoid creamy white margins. The biochemical tests, urease test, and assimilation of glucose, maltose, raffinose, and sucrose were all positive, whereas lactose assimilation was negative. All isolates were susceptible to AMB, whereas 12 isolates were susceptible to FCZ and ITZ. DOP3 was the only isolation that resisted ITZ and FCZ, with MIC values at >8 and >64 µg/mL, respectively (Table 1). Therefore, DOP3 was considered as a resistant strain *C. neoformans* and subjected to whole genome sequencing together with the non-resistant strain DOP3.1.

The genome annotation statistics by QUAST of resistant *C. neoformans* DOP3 and non-resistant *C. neoformans* DOP3.1 are shown in Table 2. The genomic size of DOP3 was 18,495 kb, while DOP3.1 was 12,373 kb. The high quality of the assembled sequences was annotated and predicted protein-coding genes. The functional information from Gene Ontology annotation is shown in data. DOP3 and DOP3.1 contained 345 and 206 contigs, respectively. The largest contig of DOP3 was 478,991 bp, and for DOP3.1 it was 480,110 bp. Total lengths of DOP3 and DOP3.1 were 18,388,727 bp and 12,391,596 pb, respectively. The percentages of G and C nucleotides of DOP3 and DOP3.1 were equal. The N50 (= the length of the shortest contig where longer and equal length contigs cover at least 50% of the assembly) of DOP3.1 was higher than for DOP3. The L50 (= count of smallest number of contigs whose length sum constitutes half of the genome length) was lower in DOP3.1.

**Figure 2:**
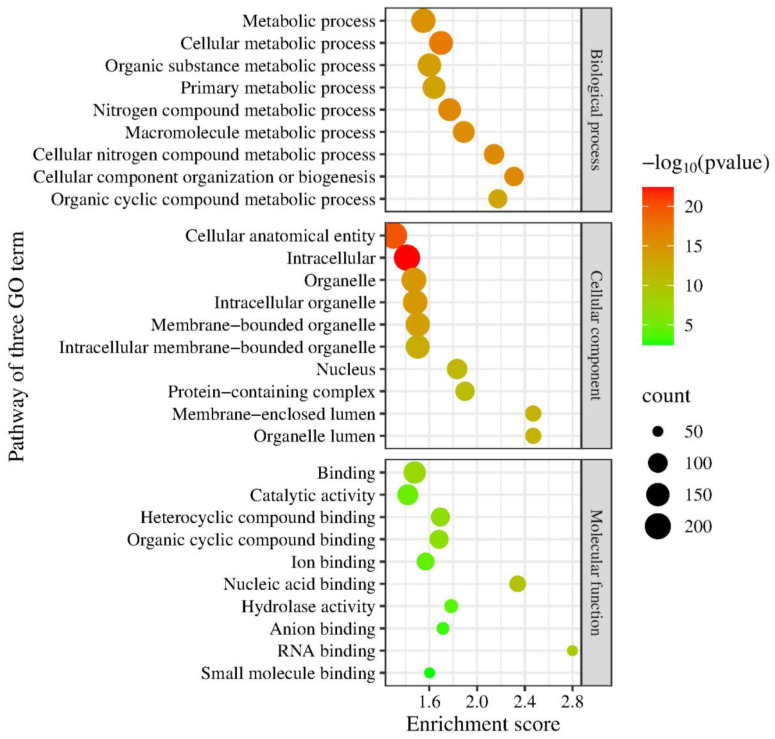
The top ten pathways of protein function annotations for three of Gene Ontology (GO term) aspects, including biological processes, cellular components, and molecular function of *C. neoformans* DOP3.1 isolates.

The functional annotation in three gene ontologies, biological processes, cellular components, and molecular function, showed the top protein pathways from the genomes of DOP3 and DOP3.1 (Figure 1). The role of *C. neoformans* DOP3 in the biological pathways consists of main target proteins in cellular, metabolic, cellular metabolic, organic substrate metabolic, primary metabolic, and nitrogen compound metabolic processes. The cellular component involved the cellular anatomical entity, intracellular, organelle, intracellular organelle, membrane-bounded organelle, and intracellular membrane-bounded organelle pathways. The molecular components included proteins in binding and catalytic activities. *C. neoformans* DOP3.1 dictated the main proteins in metabolic, cellular metabolic, organic substance metabolic, primary metabolic, and nitrogen compound metabolic processes. The main cellular component proteins were associated with the cellular anatomical entity, intracellular, organelle, intracellular organelle, and membrane-bounded organelle. The molecular function proteins were for binding and catalytic activity (Figure 2) pathways.

**Figure 3:**
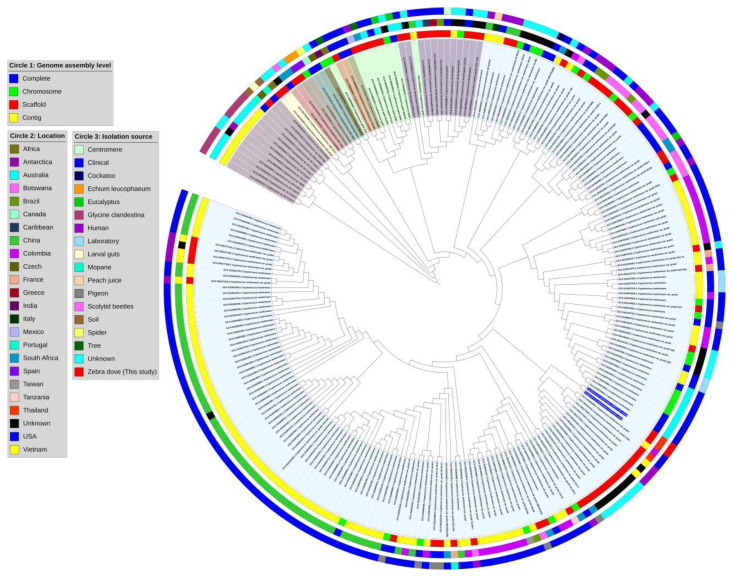
Phylogenetics tree of *C. neoformans* DOP3 and *C. neoformans* DOP3.1 isolates comparison with 230 *Cryptococcus* sp. isolates from NCBI database were in the same clade of *C. neoformans* (serotype A).

**Figure 4:**
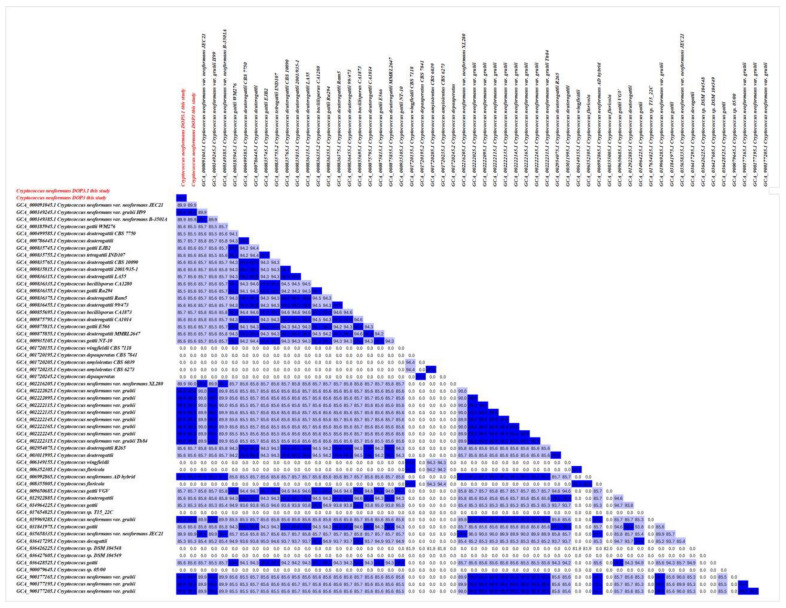
The matrix of pairwise average nucleotide identity (ANI) value of 230 *Cryptococcus* spp. annotated protein sequences and 2 of *C. neoformans* DOP3 and DOP3.1 isolates.

The phylogenetic tree of *C. neoformans* DOP 3 and DOP3.1 compared with 230 databased *Cryptococcus* isolates demonstrated that both isolates are closely related to *C. neoformans* (serotype A) in clinical samples from Spain and Thailand (Figure 3). The matrix of pairwise comparisons of ANI values of the 230 *Cryptococcus* annotated protein sequences showed 99.6% similarity to *C. neoformans* DOP3 and DOP3.1 isolates with *C. neoformans* var. *grubii* H99 (Figure 4).

**Figure 5:**
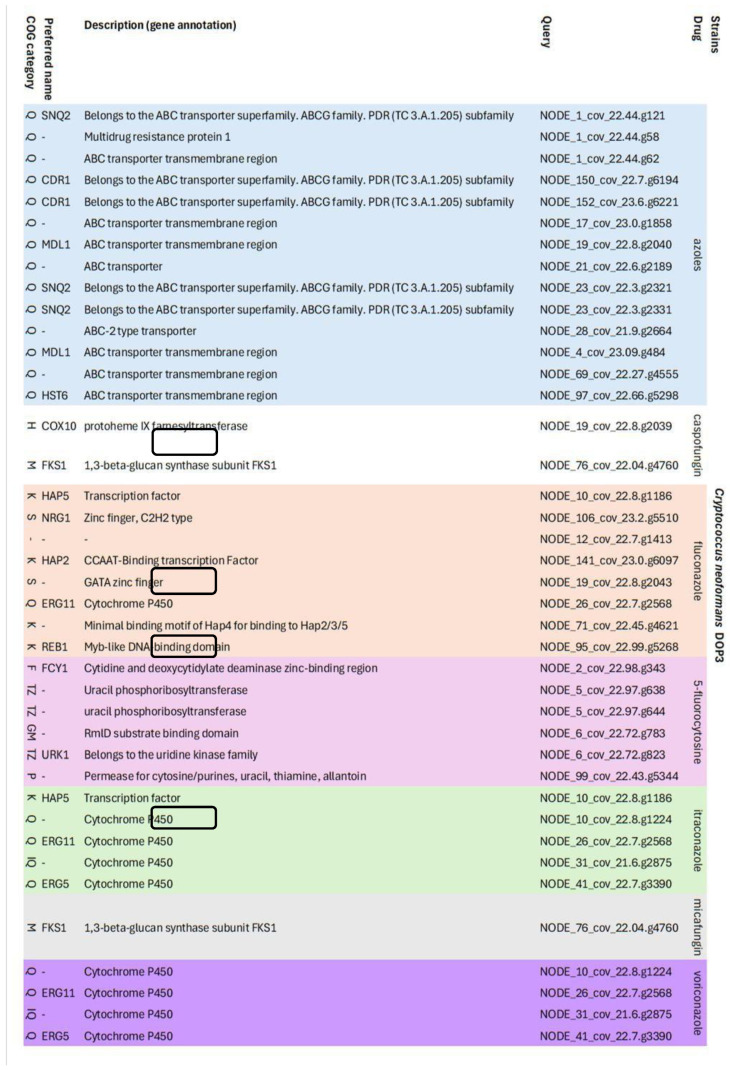
List of candidate antifungal resistance genes found after HMM analysis using the ResFungi HMM databases in resistant strain *C. neoformans* DOP3 isolates.

According to HMM analysis, *C. neoformans* DOP3 consisted of 38 candidate antifungal resistance genes against azoles (n = 14), CAS (n = 2), FCZ (n = 7), 5-FC (n = 6), ITZ (n = 4), micafungin (n = 1), and voriconazole (n = 4). The annotated genes in the azole group mostly were in the ATP-binding cassette (ABC) transporter transmembrane superfamily or regions *SNQ2, CDR1*, *MDL1*, and *HST6*. Resistance genes against FCZ were in the transcription factors (*HAP2, HAP5*), zinc finger (*NRG1*), cytochrome P450 (*ERG11*), and Myb-like DNA-binding domain (*REB1*). The most frequent resistance genes against ITZ were cytochrome P450 (*ERG5* and *ERG11*) and one gene in transcription factor *HAP5* (Figure 5).

**Figure 6:**
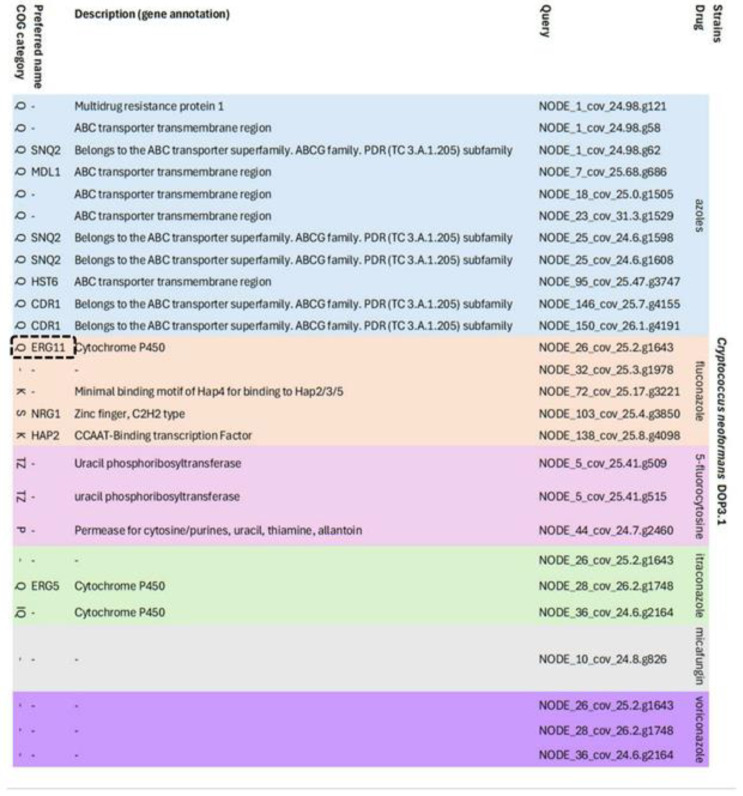
List of candidate antifungal resistance genes found after HMM analysis using the ResFungi HMM databases in non-resistant strain C. neoformans DOP3.1 isolates.

*C. neoformans* DOP3.1 had 26 candidate resistance genes against azoles (n = 11), FCZ (n = 4), 5-FC (n = 3), ITZ (n = 5), and voriconazole (n = 3). Resistance genes against the azole group belong to the ABC transporter transmembrane superfamily or regions *SNQ2*, *CDR1*, *MDL1*, and *HST6*. Resistance genes against FCZ belong to cytochrome P450 (*ERG11*), the zinc finger (*NRG1*), and CCAAT-binding transcription factor (*HAP2*). Resistance genes belonging to cytochrome P450 (*ERG5*) were found against ITZ (Figure 6).

## 4. Discussion

This research is the first report of *C. neoformans* (serotype A) from zebra dove excreta in Thailand. This variety is widespread in the environment, where it is primarily associated with pigeon excreta [38]. Genotypic analyses of serotype A have identified three genetically isolated subpopulations, VNI, VNII, and VNB [39]. *C. neoformans* var. *grubii* is responsible for the deadly meningoencephalitis correlated with HIV status [40,41]. The *C. neoformans* var. *grubii* isolates from 11 provinces of Thailand were highly homogenous, consisting of the same genetic background (globally known as VNI) and exhibiting only ten nearly identical sequence types (STs), with three (STs 44, 45, and 46) dominating [42]. The genome sizes of the resistant strain *C. neoformans* DOP3 and the non-resistant strain *C. neoformans* DOP3.1 isolate were 18 and 12 Mb with 14 chromosomes. Most isolates of *C. neoformans* have a genome size of approximately 19 Mb with 14 chromosomes [43].

Antifungal susceptibility tests showed all 13 isolates in our study were highly sensitive to AMB (100%), followed by fluconazole and itraconazole (98% each). Similarly, *C. neoformans* in pigeon excreta from eastern Thailand displayed sensitivity to AMB, FCZ, and ITZ [44]. However, our research found that *C. neoformans* DOP3 showed high resistance to FCZ (MIC >64 µg/mL) and moderate resistance to ITZ (MIC >8 µg/mL). A previous study [45] determined the principal genes associated with resistance to four classes of drugs: ERG11, AFR1, MSH2, and YAP1 for resistance against FCZ; ERG11 and HOB1 for resistance against AMB; FCY1, FCY2, FUR1, UXS1, URA6, UGD1, and NRG1 for resistance against 5-FC; and FKS1 and CDC50 for resistance against CAS.

Insight into the genome of *C. neoformans* DOP3 indicates it encodes the important genes ERG11 for resistance to FCZ and ITZ, FCY1 for resistance to 5-FC, and FKS1 for resistance to CAS. Therefore, it exhibited a resistant phenotype as indicated by high MIC values against FCZ and ITZ. Similar to a previous study [46], *C. neoformans* var. *grubii* isolated from a cat had multiple azole-resistant strains, including one resistant to FCZ, which encoded a G344S substitution in Erg11p. Clinical isolates of *C. neoformans* var. *grubii* in China were wild type to voriconazole and 89.15% were non-wild-type to AMB, with eight isolates resistant to FCZ [47]. In contrast, ERG11 overexpression and variations in ERG11 coding sequences were not responsible for the high MIC values against azole observed for *C. gattii* strains from the Pacific Northwest of the USA [48]. The presence of FCY1 genes in *C. neoformans* DOP3.1 may cause high-level resistance to 5-FC consistent with previous reports [49]. The FKS1 gene of *C. neoformans* is essential for viability [50]. *C. neoformans* tolerance to extremely low levels of FKS1 expression led to poor activity of 1,3-β-glucan synthase inhibitors that activate the cell wall integrity stress response and increase susceptibility to CAS [51]. *Cryotococcus neoformans* as a pathogen in humans is intrinsically resistant to CAS due to the β-1,3-glucan synthase encoded by FKS1 [52]. The search for resistance genes in the non-resistant strain *C. neoformans* DOP 3.1 found only the gene ERG11 for resistance to ITZ. Therefore, *C. neoformans* DOP 3.1 did not show any phenotypic resistance in antifungal susceptibility testing.

Normally, the treatment of *C. neoformans var. grubii* infection (cryptococcal meningitis) in humans follows the guidelines of the Society for Infectious Diseases of America [22] and the World Health Organization [53]. The treatment consists of three stages. The induction stage is treatment with AMB (0.7–1.0 mg/kg/day) together with 5-FC (100 mg/kg/day) for two weeks. The consolidation stage is treatment with FCZ (400 mg/day) for 8 weeks, followed by the maintenance stage with FCZ (200 mg/day) for 6–12 months. According to our research, there exists a strain of *C. neoformans* resistant to VCZ. A previous study [54] reported the rates of FCZ resistance seen in *C. neoformans* var. *grubii*, especially among *C. neoformans* ST5 isolates. Therefore, FCZ use in the consolidation and maintenance stages should be considered for replacement or the drug should be used in combination with other antifungal products. Moreover, farm sanitation should be strictly applied, and immunocompetent people should avoid contact with zebra dove excreta.

## Figures and Tables

**Table 1:** The minimum inhibitory concentration (MIC) and minimum fungicidal concentration (MFC) value for amphotericin B, fuconazole, and itraconazole tested against 13 *Cryptococcus* isolates and *Candida albicans* as a control.

No.	Isolate	Amphotericin B (µg/mL)			Fluconazole (µg/mL)			Itraconazole (µg/mL)		
		MIC		MFC	MIC		MFC	MIC		MFC
Control	*C. albicans* (ATCC 90028)	0.125			0.5			0.03		
1	Tip3	0.03	S	0.03	4	S	8	0.25	SDD	0.25
2	Rod5	0.03	S	0.03	4	S	8	0.25	SDD	0.25
3	DOP2	0.0625	S	0.25	4	S	8	0.25	SDD	0.25
4	DOP3	0.125	S	0.125	>64	R	>64	>8	R	0
5	DOP3.1	0.03	S	0.125	4	S	16	0.125	S	0.125
6	DOP3.2	0.03	S	0.0625	4	S	4	0.125	S	0.125
7	DOP12.1	0.0625	S	0.125	4	S	8	0.125	S	0.25
8	DOP12.2	0.03	S	0.125	4	S	8	0.125	S	1
9	DOP17	0.03	S	0.125	4	S	8	0.125	S	0.25
10	K3	0.125	S	0.25	4	S	4	0.25	SDD	0.5
11	B3	0.03	S	0.125	4	S	8	0.125	S	0.25
12	TL12.1	0.0625	S	0.125	4	S	8	0.25	SDD	0.25
13	TL12.2	0.0625	S	0.125	4	S	4	0.25	SDD	0.25

S = susceptible; R = resistant, SDD = susceptible-dose-dependent, MIC = minimum inhibitory concentration, MFC= minimum fungicidal concentration.

**Table 2:** The quality assessment results of the assembled genomes of the resistant isolate *C. neoformans* DOP3 and non-resistant isolate *C. neoformans* DOP3.1 in FASTA format by QUAST v.5.0.2.

Genome Features	Resistant Isolate *(C. neoformans* DOP3)	Non-Resistant Isolate *(C. neoformans* DOP3.1)
Contigs (≥0 bp)	716	433
Contigs (≥1000 bp)	304	178
Total length (≥0 bp)	18,495,918	12,457,094
Total length (≥1000 bp)	18,360,890	12,373,593
Genome size (kb)	18,495	12,373
Contigs	345	206
Largest contigs (bp)	478,991	480,110
Total length (bp)	18,388,727	12,391,596
GC content (%)	48.19	48.20
N50 (bp)	153,969	170,860
N90 (bp)	45,564	48,649
auN (bp)	172,572.5	184,709.8
L50	39	25
L90	128	78

## Data Availability

The datasets used and/or analyzed during the current study are available from the corresponding author on reasonable request.
